# Brassicasterol from Edible Aquacultural *Hippocampus abdominalis* Exerts an Anti-Cancer Effect by Dual-Targeting AKT and AR Signaling in Prostate Cancer

**DOI:** 10.3390/biomedicines8090370

**Published:** 2020-09-22

**Authors:** Yinzhu Xu, Sooin Ryu, You-Kyung Lee, Hyo-Jeong Lee

**Affiliations:** 1 Department of Science in Korean medicine, College of Korean Medicine, Graduate School, Kyung Hee University, Hoegi-dong, Dongdaemun-gu, Seoul 02453, Korea; xyz3402@khu.ac.kr; 2 Department of Food Science & Services, Eulji University, Seongnam, Kyungido 13135, Korea; ysicute@euji.ac.kr; 3 Department of Cancer Preventive Material Development, College of Korean Medicine, Graduate School, Kyung Hee University, Hoegi-dong, Dongdaemun-gu, Seoul 02453, Korea; yklee9575@khu.ac.kr

**Keywords:** prostate cancer, seahorse, *Hippocampus abdominalis*, brassicasterol, AR, AKT

## Abstract

In the Compendium of Materia Medica, seahorse (*Hippocampus*) is considered effective for the reinforcement of kidney and men’s health. However, the role of seahorse on human health lacks scientific evidence. Therefore, we evaluated the effect of seahorse on human prostate cancer using various in vitro methods and identified bioactive compound. Seahorse lipid extract (SHL) decreased androgen receptor (AR) and prostate-specific antigen (PSA) expression in dihydrotestosterone (DHT)-induced LNCaP cells of prostate cancer. Gas Chromatography (GC)-mass spectrometry data showed that brassicasterol was present in *H. abdominalis*. Brassicasterol downregulated the expression of AR and PSA in DHT-induced LNCaP cells. Brassicasterol induced apoptosis accompanied by sub-G1 phase arrest and inhibited migration in LNCaP cells. We confirmed that AKT and AR mediated the anti-cancer effect of brassicasterol using siRNA transfection. Brassicasterol exerts an anti-cancer effect in AR-independent cancer as well as in AR-dependent cells by AKT inhibiting. Our findings suggest that SHL has the anticancer potential via inhibition of AR and demonstrated that brassicasterol from *H. abdominalis* exerted an anti-cancer effect by dual-targeting AKT and AR signaling in prostate cancer.

## 1. Introduction

Prostate cancer is the second most frequent malignancy in men worldwide. The incidence and mortality rate of prostate cancer globally are strongly correlated to increasing age (over 65 years of age) [1]. Prostate cancer affects the prostate, a gland in the male reproductive system that produces seminal fluid that nourishes and transports sperm [2]. Prostate cancer can cause symptoms such as lower urinary tract symptoms, frequent urination, nocturia, hematuria, and dysuria. The androgen receptor (AR) plays a role in sexual and physiological development, especially development of the prostate in men [3]. The binding of androgen to AR initiates the signal of cell growth and proliferation in cancer [4,5,6]. Prostate-specific antigen (PSA) is one of the genes regulated by AR and is considered the most sensitive biomarker for confirming the existence of prostatic disease and prostate cancer [7]. Therefore, androgen-deprivation therapy is the first-line treatment for prostate cancer. However, long-term androgen-deprivation therapy can cause the progression of castration-resistant prostate cancer (CRPC) [8]. CRPC tumors sustain the expression of AR and its regulated genes, indicating that AR signaling continues to function [9,10,11,12]. CRPC tumors show advanced AR activation, including AR gene amplification, gain-of-function mutations [13,14], alterations in expression and function of crucial AR co-regulators, and generation of ligand-binding domain truncated AR splice variants(AR-Vs) [15]. These data indicate that targeting AR is an important therapy in prostate cancer.

The phosphatidylinositol 3-kinase (PI3K)/AKT/mammalian target of rapamycin (mTOR) signaling is often activated and highly expressed in PCa and play a leading role in CRPC progression and resistance to drug-induced apoptosis [16,17]. 42% of primary prostate cancers and 100% of metastatic cancers are caused by genetic changes of elements of PI3K/AKT/mTOR pathway [16]. It has been proved that correlative feedback activation of PI3K/AKT and AR signaling pathways let cancer cells to use to one pathway for survival when the other pathway is pharmacologically blocked [18,19]. These findings support that co-targeting both pathways may fulfill better results for CRPC patients.

In alternative medicine, seahorse has been used for improving sexual function and curing infertility, baldness, asthma, and arthritis, but indiscriminate overfishing has made it an endangered species. In 2015, *Hippocampus abdominalis* was approved as a food product in Korea, resulting in successful aquaculture.

Seahorse has various biomedical benefits. Seahorse has been reported to have anti-cancer [20,21], antimicrobial [22], anti-rheumatism [23,24], anti-oxidant [25,26], anti-inflammation [27], benign prostatic hyperplasia alleviative [28], and neuroprotective effects [29,30]. The two studies for anticancer effect of seahorse showed bioactive compounds and peptide from seahorse and their anti-metastatic ability in HT 1080 human fibrosarcoma cells. However, the mechanism of action of seahorse in prostate cancer has not yet been elucidated.

In the present study, we evaluated *H. abdominalis* lipid extract (SHL) and its sterol on anti-cancer ability in prostate cancer cells. Lipid extract has abundant sterol as expected. Therefore, we checked brassicasterol, which is a phytosterol found in marine algae, fish, and shellfish, using GC/MS analysis.

Brassicasterol is a 28-carbon sterol synthesized by several unicellular algae (phytoplankton) and some terrestrial plants such as rape. Brassicasterol is a phytosterol and is known for lowering the serum cholesterol level, leading to cardiologic health benefits [31,32]. Brassicasterol is not known for its anti-cancer effect in prostate cancer.

In the present study, we showed the anti-cancer effects of *H. abdominalis* and its bioactive compound, brassicasterol, on AR and AKT expression in prostate cancer cells.

## 2. Experimental Section

### 2.1. Preparation of SHL

The blended 100 g of frozen seahorse (*H. abdominalis*, Seahorse Australia, Beauty Point, TAS, Australia) was homogenized with 300 mL of chloroform/methanol (2:1) for 3 min. The homogenized sample was filtered. The residue was homogenized in 100 mL chloroform and filtered again. KCl (0.88%, 100 mL) was added to the filtrate and incubated for 24 h in the dark at 4 °C. Anhydrous sodium sulfate (5 g) was added to remove the excess of water in the chloroform layer and incubated in the dark at 4 °C for 30 min. Only the chloroform layer was filtered and concentrated under reduced pressure at 40 °C with a vacuum evaporator to obtain lipid. The lipid extract was dissolved in chloroform, filtered, and purged. Methyl hydroxylamine chloride (50 µL) in pyridine solution and 50 µL of BSTFA in 1% TMCS were added to the vial where all moisture was removed and allowed to react at 65 °C for 60 min. Brassicasterol (Sigma-Aldrich, St. Louis, MO, USA) was prepared at five different concentrations (1, 5, 10, 20, 50, and 100 mg/L) for the preparation of calibration standards.

### 2.2. Gas Chromatography-Mass Spectrometry (GC-MS)

We performed Gas Chromatography-Mass Spectrometry (GC-MS) analysis to identify brassicasterol from seahorse using an ISQ LT gas chromatograph interfaced with a single quadrupole mass spectrometer (Thermo Scientific, Asutin, TX, USA). GC-MS analysis was performed on a DB-5MS column (60 m × 0.25 mm inner diameter, 0.25 mm film thickness, Agilent Technologies, Santa clara, CA, USA). It started at 325 °C for 3 min, gradually increasing the oven temperature (320 °C at 10 °C /min, 330 °C at 2 °C /min (held for 8 min), 380 °C at 30 °C /min, and held for 3 min). N_2_ was used as a carrier gas. The MS detection system included an electron impact ionization. Total running time was 43 min. The injection volume was 1 µL at 280 °C. The energy of the electron impact ionization was 70 eV.

### 2.3. Cell Culture

LNCaP and PC-3 human prostate cancer cell lines from the Korean cell bank (KCLB) (Seoul, Korea) were used for this study. The cells were cultivated in RPMI-1640 medium with 10% fetal bovine serum (FBS), 2 μM l-glutamine, and penicillin/streptomycin (WelGene, Deagu, Korea) in a cell incubator (Thermo Scientific, Asutin, TX, USA) brassicasterol (Sigma-Aldrich, St. Louis, MO, USA) was used for in vitro assays and mechanism study.

### 2.4. Cell Viability Assay

We measured cell viability using AKT siRNA and brassicasterol in LNCaP and PC-3 cells using the CELLOMAX™ viability kit based on the tetrazolium salt (2-(2-methoxy-4-nitrophenyl)-3-(4-nitrophenyl)-5-(2,4-disulfophenyl)-2H-tetrazolium, monosodium salt (WST-8) (Precaregene, Hanam, Kyungido, Korea). The control and AKT siRNA (1 pmol) and brassicasterol (10 μM) were added to 1 × 10^4^ cells per well in a 96-well plate for 48 h. CELLOMAX™ reagent (10 μL) was added and incubated for 2 h at 37 °C in the dark. A microplate reader (Tecan, Sunrise, Männedorf, Switzerland) was used for measuring the optical density (O.D) at 450 nm. Cell viability equation:

Cell viability (%) = (O.D (test sample) − O.D (blank)) / (O.D (control) − O.D (blank)) × 100.(1)

### 2.5. Western Blotting

RIPA buffer (1% NP-40, 150 mM NaCl, 50 mM Tris-HCl, pH 7.4, 0.25% sodium deoxycholate, 1 mM Na_3_VO_4_, 1 M EDTA, 1 mM NaF, and protease inhibitor cocktail) was used for extraction protein. The lysates were quantified (Bio-Rad DC protein assay kit II; Bio-rad, Hercules, CA, USA), separated proteins on SDS-PAGE gels (8–10%), and transferred the molecules to a nitrocellulose membrane (Amersham Pharmacia, Uppsala, Sweden). The transferred membrane was blocked with 5% non-fat skim milk and incubated with primary antibody for β-actin (Sigma-Aldrich, St. Louis, MO, USA), PSA (Dako, Santa Clara, CA, USA), phosphor-AKT, AKT, PARP (Santa Cruz Biotechnology, Dallas, Texas, USA), AR, E-cadherin, Vimentin, and cleaved caspase-3 (Cell Signaling, Beverly, MA, USA). Horseradish peroxidase (HRP)-conjugated anti-rabbit or mouse secondary antibodies were added and incubated to the membrane. Enhanced chemiluminescence (ECL) Western blotting substrate (Amersham Pharmacia, Uppsala, Sweden) was used to detect HRP enzyme activity.

### 2.6. RNA Extraction and qRT-PCR

Total RNA was isolated from cells using TRIzol (Invitrogen, Carlsbad, CA, USA). cDNA was synthesized from the purified total RNA using the High-Capacity cDNA Reverse Transcription kit (Promega, Madison, WI, USA). qRT-PCR was performed using the SYBR green RT-PCR kit (Bioneer, Seoul, Korea) and custom-designed primers (AR Forward 5′-CC TGG CTT CCG CAA CTT ACA C-3′, Reverse 5′-GG ACT TGT GCA TGC GGT ACT CA-3′ and β-actin Forward 5′-AA GAG AGG CAT CCT CAC CCT-3′, Reverse 5′-AT CTC TTG CTC GAA GTC CAG-3′) on the Thermal Cycler Dice^®^ Real Time System III (Takara Bio Inc., Shiga, Japan).

### 2.7. Fluorescence-Activated Cell Sorting (FACS) Analysis

The cells which were treated with brassicasterol were fixed with 70% ethanol. After fixing, RNAase A (10 mg/mL) was added to the cells and incubated for 1 h at 37 °C. Then, the cells were stained with propidium iodide (PI) (50 μg/mL). After filtering the cells using a nylon mesh, the DNA content of stained cells was analyzed using the CellQuest Software (BD Biosciences, San Jose, CA, USA) on a FACS caliber flow cytometer (Becton Dickinson, Franklin Lakes, NJ, USA).

### 2.8. Wound-Healing Assay

The LNCaP cells (1 × 10^6^ cells/mL) were seeded in a 6-well plate and incubated at 37 °C. When the cells reached 70% confluence, they were scratched using a 200-μL pipette tip, followed by washing with phosphate-buffered saline (PBS). Brassicasterol was treated to the cells for 24 h. Then, the cells were fixed and stained with Diff-Quick solution kit. The images were taken a photo under a light microscope (Nikon, Tokyo, Japan). The number of cells that migrated to the scratched empty area was counted.

### 2.9. 3D Tumor Organoids

For the generation of the LNCaP tumor organoids, cells were seeded into 96-well round bottom ultra-low attachment plates (Corning, Corning, NY, USA) at 2000 viable cells per well. The LNCaP spheroids were grown in RPMI medium with 3% FBS. The plates were incubated for 5 days at 37 °C. Five days after the spheroids formed, 100 μg/mL brassicasterol was added to the formed spheroids for 48 h. For apoptosis analysis, 2 μM CellEvent (Invitrogen) was added to each well and incubated for 1 h. Pictures were obtained using a fluorescence microscope (Nikon).

### 2.10. siRNA Transfection

The AR, control, and AKT siRNA were purchased from Bioneer (Daejeon, Korea). LNCaP and PC-3 cells were plated at a density of 5 × 10^4^ cells per well in a 6-well plate. Cells were transfected using siRNA (25 pmol/final siRNA used per well) with siRNA transfection reagent (Polyplus-transfection, Illkirch, France) for 48 h. After treatment, cells were stimulated for Western blotting and stained using crystal violet. The O.D was measured using a microplate reader (Tecan, Männedorf, Switzerland) at 570 nm (crystal violet).

### 2.11. Crystal Violet Staining and Cell Growth Assay

To investigate the anti-proliferative effect of brassicasterol, crystal violet staining was used. Brassicasterol were treated different concentrations (0, 10, and 50 μM) for 5 days to LNCaP cells (1 × 10^5^ cells) seeded in a 6-well plate. After 5 days, the cells were fixed with 1% glutaraldehyde (JUNSEI, Tokyo, Japan). Then, 0.05% crystal violet (Sigma-Aldrich) was put for 30min to stain the cells. After washing with deionized water and drying, A 70% ethanol solution was used to restain the crystal violet. A microplate reader (Tecan) was used for measuring optical density (O.D) at 570 nm.

### 2.12. Statistical Analyses

The data were expressed as means ± standard deviation (SD) of three replicates per experiment. Analysis of variance (ANOVA) was conducted to determine the significance of differences between groups; *p* < 0.05 was considered significant.

## 3. Results

### 3.1. Seahorse Lipid Extract (SHL) Inhibits Androgen Receptor (AR) Expression in Dihydrotestosterone (DHT)-Induced LNCaP Cells

**Figure 1:**
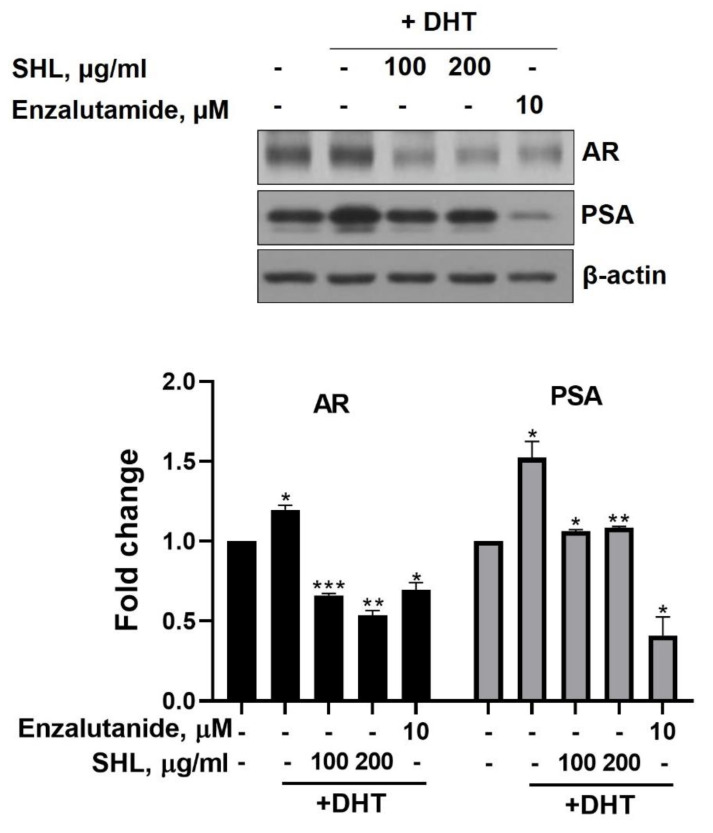
The inhibitory effect of *Hippocampus abdominalis* (seahorse) lipid extract (SHL) on androgen receptor (AR) and prostate-specific antigen (PSA) expression in DHT-induced LNCaP cells. LNCaP cells were treated with DHT (2 nM) and SHL (100 and 200 μg/mL) in RPMI 1640 media with 5% charcoal-stripped serum for 24 h; then, the cells were lysed to do Western blotting assay for AR and PSA expression. Bar graph represents the quantification of interest protein related to β-actin, presents as a fold change of control. (*) *p* < 0.05; (**) *p* < 0.01; (***) *p* < 0.001.

We examined the effect of SHL on AR protein expression levels in DHT-induced LNCaP cells. DHT-induced LNCaP cells showed high AR (1.2-fold) and PSA (1.5-fold) expression compared to that of the control (Figure 1). SHL inhibited AR and PSA expression at both 100 and 200 μg/mL concentrations in DHT-induced LNCaP cells (Figure 1). The AR expression of SHL (100 μg/mL, 0.66-fold; 200 μg/mL, 0.54-fold) was similar to that of enzalutamide (0.69-fold), which is an anti-cancer agent and AR inhibitor (Figure 1).

### 3.2. Identification of Brassicasterol from SHL Using GC-MS

**Figure 2:**
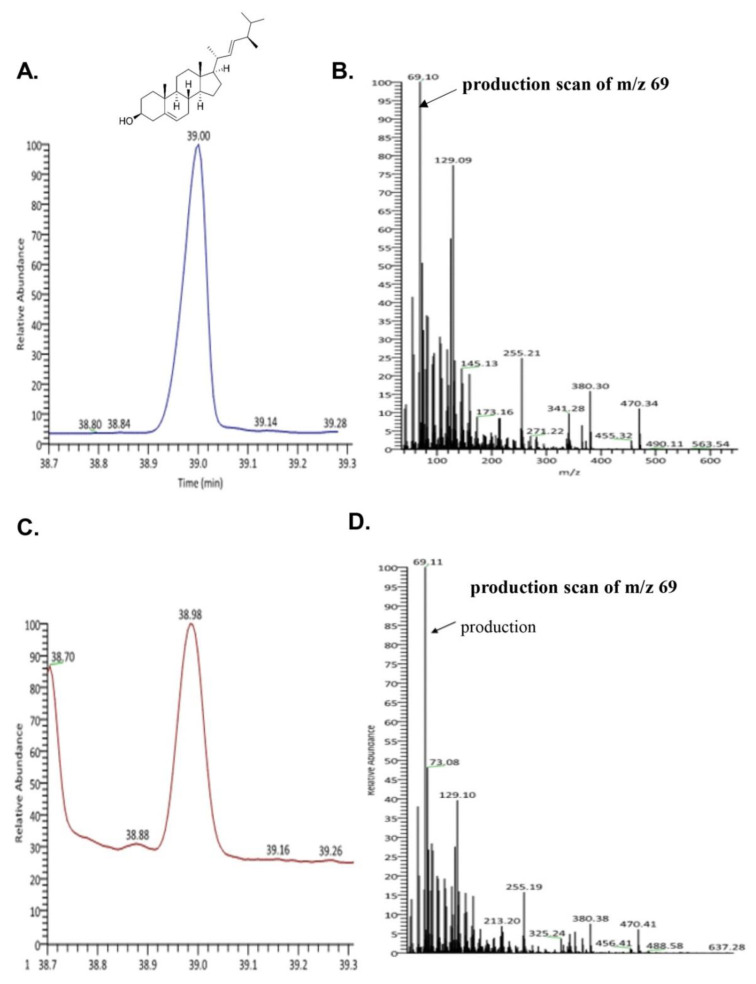
Ion chromatogram of trimethylsilyl derivatives of acidic components and ass spectra by GC/MS analysis of (**A**) total ion chromatogram of brassicasterol; (**B**) mass spectra of brassicasterol; (**C**) total ion chromatogram of lipid extraction from *Hippocampus abdominalis* (SHL); (**D**) mass spectra of lipid extraction from *Hippocampus abdominalis* (SHL).

To evaluate the bioactive compound from seahorse, we performed GC-MS. We confirmed the existence of brassicasterol in SHL. As shown in Figure 2C,D, the retention time and mass spectrum of SHL (38.98 min, m/z 69.11) were consistent with those of brassicasterol (elution time: 39 min, m/z 69.10) (Figure 2A,B). SHL contained 35.5 mg of brassicasterol per 100 g.

### 3.3. Brassicasterol Inhibits AR and PSA Expression in LNCaP Cells

**Figure 3:**
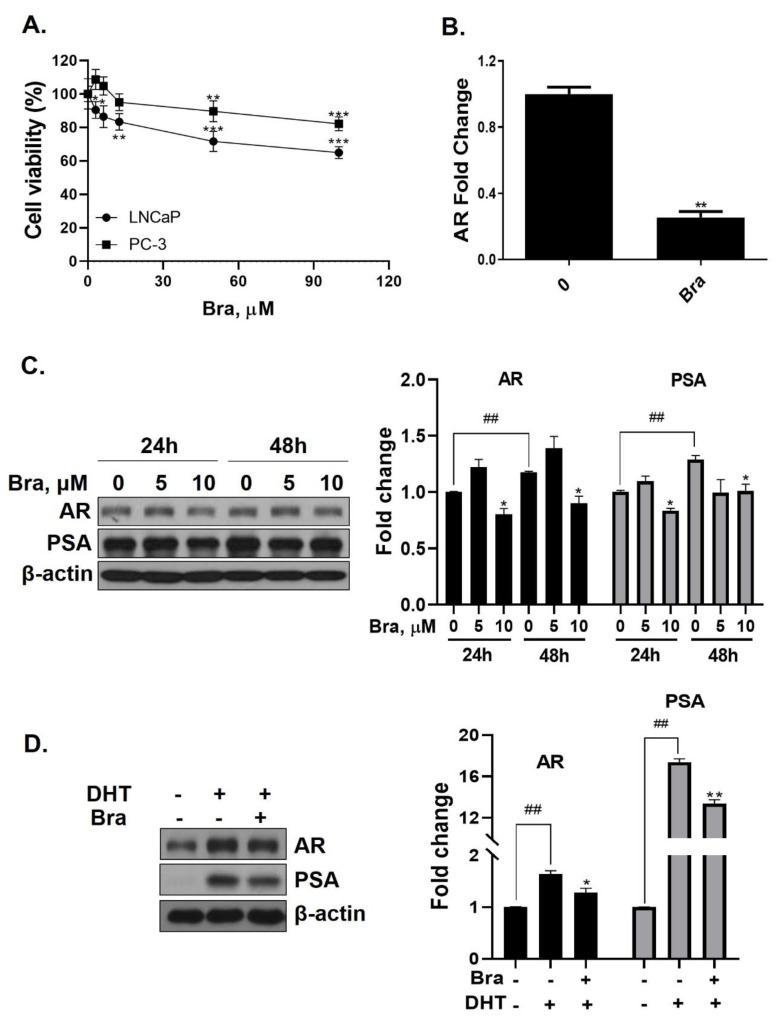
Inhibitory effect of brassicasterol on AR and PSA expression in LNCaP cells. (**A**) Cell viability assay. LNCaP and PC-3 cells were treated with various concentrations of brassicasterol for 24 h. (*) *p* < 0.05, (**) *p* < 0.01, and (***) *p* < 0.001 (in comparison to control). (**B**) LNCaP cells were treated with 10 μM brassicasterol for 24 h. AR mRNA levels using qRT-PCR. Quantitative mRNA levels are shown. (**) *p* < 0.01 (in comparison to control). (**C**) Changes of the AR and PSA expression in LNCaP cells by treatment with brassicasterol according to concentration and time. Bar graph represents the quantification of interest protein related to β-actin, presents as a fold change of control. (*) *p* < 0.01 (in comparison to control of each time) (##) *p* < 0.01(in comparison to 24h control). (**D**) Changes of AR and PSA expression in DHT-induced LNCaP cells by treatment with brassicasterol. Bar graph represents the quantification of interest protein related to β-actin, presents as a fold change of control. (*) *p* < 0.05, (**) *p* < 0.01 (in comparison to DHT-stimulated control) (##) *p* < 0.01 (in comparison to control).

Cell viability of brassicasterol was analyzed in LNCaP and PC-3 cells. LNCaP or PC-3 cells (1 × 10^4^ cells/well) were treated with various concentrations of brassicasterol for 24 h. As shown in Figure 3A, the viability of LNCaP cells and PC-3 cells was reduced to 36% and 18%, respectively, when incubated with 100 μM of brassicasterol. Brassicasterol had more affective in LNCaP cells than PC-3 cells. We examined the AR mRNA expression in LNCaP cells to investigate whether brassicasterol isolated from SHL had an AR inhibitory effect like that of SHL. Brassicasterol (10 μM) was added to LNCaP cells for 24 h, and the mRNA levels of AR were examined using quantitative reverse transcription polymerase chain reaction (qRT-PCR) (Figure 3A). Brassicasterol suppressed the AR mRNA level in LNCaP cells (Figure 3B).

The change in AR and PSA protein levels induced by brassicasterol was measured in time and dose course using Western blotting. The control group showed a time-dependent increase in both AR (1.17-fold increase) and PSA (1.2-fold increase) expression (Figure 3C). Brassicasterol (10 μM) inhibited PSA and AR expression at both 24 and 48 h (Figure 3C). The AR expression of 10 μM brassicasterol for 24 and 48 h treatment was indicated 0.80-fold (24 h control, 1-fold)and 0.89-fold (48 h control, 1.17-fold), respectively. To validate the decrease in AR and PSA expression induced by brassicasterol, brassicasterol was added to DHT-induced LNCaP cells. DHT-induced LNCaP cells showed upregulated AR and PSA protein expression levels. Brassicasterol inhibited the upregulated AR and PSA expressions in DHT-induced LNCaP cells (Figure 3D).

### 3.4. Brassicasterol Inhibits Cell Growth and Induces Sub-G1 Phase Arrest in LNCaP Cells

**Figure 4:**
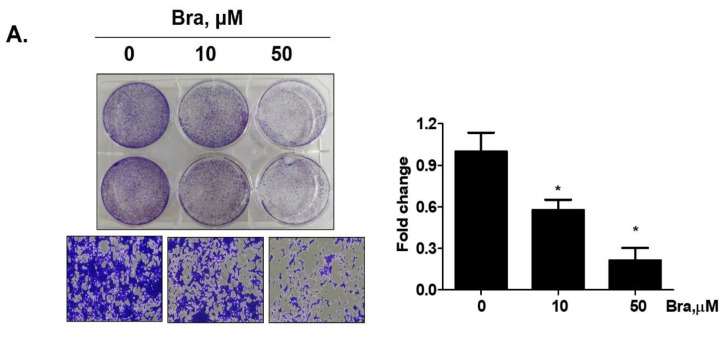

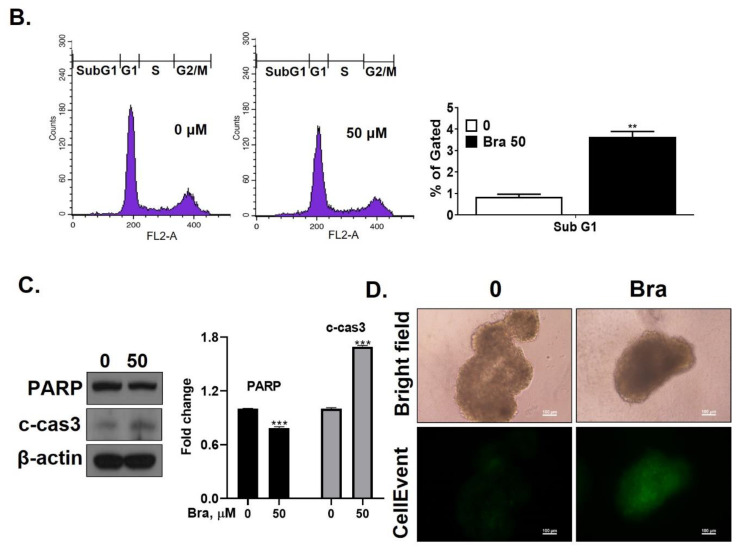
Inhibitory effect of brassicasterol on cell growth in LNCaP cells. (**A**) 10 and 50 μM of Brassicasterol treated to LNCap Cells for 5 days. The cells were stained with a crystal violet staining solution, and randomly chosen were photographed and resolved in 70% EtOH, and the absorbance was measured using a microplate reader. Data represent mean ± SD. (*) *p* < 0.05 compared with control. (**B**) Cell cycle analysis of brassicasterol in LNCaP cells. The LNCaP cells were treated with 50 μM of Brassicasterol for 48 h and analyzed by flow cytometry. Bar graphs showed the quantification of subG1 (%). (**) *p* < 0.01 compared with control. (**C**) Brassicasterol-treated (50 μM, 48 h) LNCaP cell lysates were prepared and subject to Western blotting for apoptosis makers (PARP and cleaved caspase-3). Bar graph represents the quantification of interest protein related to β-actin, presents as a fold change of control. (***) *p* < 0.01 (in comparison to control). (**D**) Inhibitory effect of brassicasterol on LNCaP 3D tumor organoids growth. 5 days after formed spheroid, 50 μM brassicasterl was treated to the formed spheroids (*n* = 6 /group) for 48 h.

Brassicasterol (10 or 50 μM) was added to LNCaP cells for 5 days to determine whether it inhibited cancer cell proliferation after prolonged exposure. As shown in Figure 4A, brassicasterol decreased the number of LNcaP cells in a concentration-dependent manner. 50 μM brassicasterol exhibited 79% cell growth inhibitory effect (IC_50_ = 18.43 ± 0.052 μM).

We checked whether a 48 h treatment with 50 μM brassicasterol affected the cell cycle in LNCaP cells. After 48 h treatment with 50 μM brassicasterol, the sub-G1 phase was weakly arrested (Figure 4B). We performed Western blotting to confirm whether the induction of sub-G1 phase by brassicasterol was associated with the expression level of apoptosis-related proteins (PARP and cleaved caspase-3). Brassicasterol inhibited PARP expression and induced cleaved caspase-3 expression (Figure 4C). To assess the effect of brassicasterol on tumor growth, we used LNCaP tumor organoids (Figure 4D). The three-dimensional culture model mimics some aspects of the in vivo tumor organization and microenvironment, providing a better understanding of the response of the cells to the drug. We confirmed that brassicasterol induced apoptosis using CellEvent, a fluorogenic caspase-3/7 substrate. As shown in Figure 4D, brassicasterol induced apoptosis in LNCaP cell spheroids.

### 3.5. AR Mediates Brassicasterol-Induced Suppression of Cell Migration and EMT in LNCaP Cells

**Figure 5:**
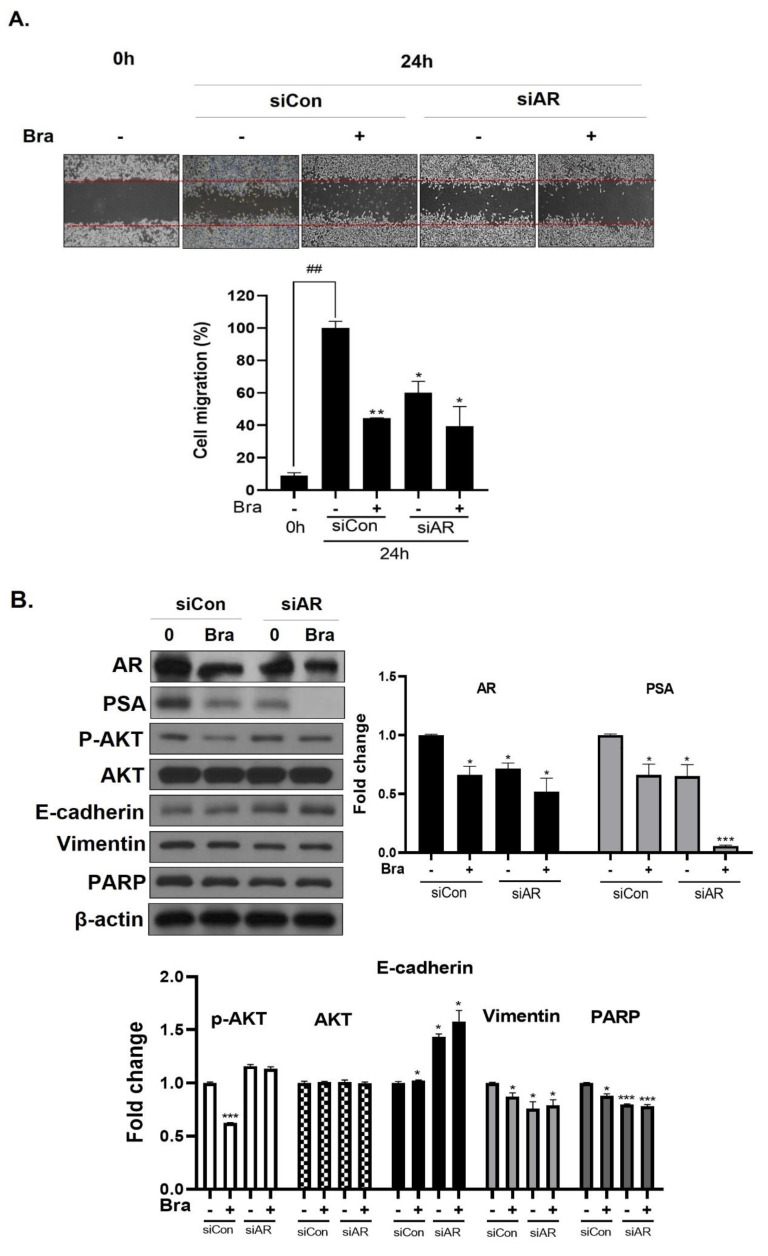
Effect of AR siRNA on cell migration, proliferation, and apoptosis-related markers in Brassicasterol treated LNCaP cells. LNCaP cells were transfected with AR siRNA for 24 h and were incubated in the presence or absence of brassicasterol (10 μM) for 24 h. (**A**) A wound-healing assay assessed cell migration. Bar graph represents the quantification of cell migration, present as a percentage of control of siRNA. (*) *p* < 0.05, (**) *p* < 0.01 (in comparison to 24 h control of siRNA) (##) *p* < 0.01 (in comparison to 0h control). (**B**) The cell lysates were prepared and subjected to Western blotting to determine the expression of AR, PSA, p-AKT, AKT, E-cadherin, vimentin, PARP, and β-actin. Bar graphs represent the quantification of interest protein related to β-actin, present as a fold change of control of siRNA. (*) *p* < 0.05, (***) *p* < 0.01 (in comparison to control).

To assess whether brassicasterol-mediated cell migration, including EMT, were dependent on AR inhibition, we examined the effect of AR knockdown on migration in LNCaP cells. AR knockdown by small interfering RNA (siRNA) enhanced brassicasterol-mediated cell migration as determined using a wound-healing assay (Figure 5A). AR siRNA showed 40% cell migration inhibitory effect. Brassicasterol showed 54% cell migration inhibitory effect. We assessed whether these changes were associated with the regulation of the expression of cell proliferation and cell EMT-mediated migration regulatory proteins. AR siRNA inhibited PSA, vimentin, and PARP expression and induced E-cadherin expression. AR siRNA contributed to the anti-cancer effects of brassicasterol in LNCaP cells. However, AR knockdown did not affect the expression of phospho-AKT, as shown in Figure 5B.

### 3.6. AKT Mediates Brassicasterol-Induced Suppression of the AR Signaling Pathway

**Figure 6:**
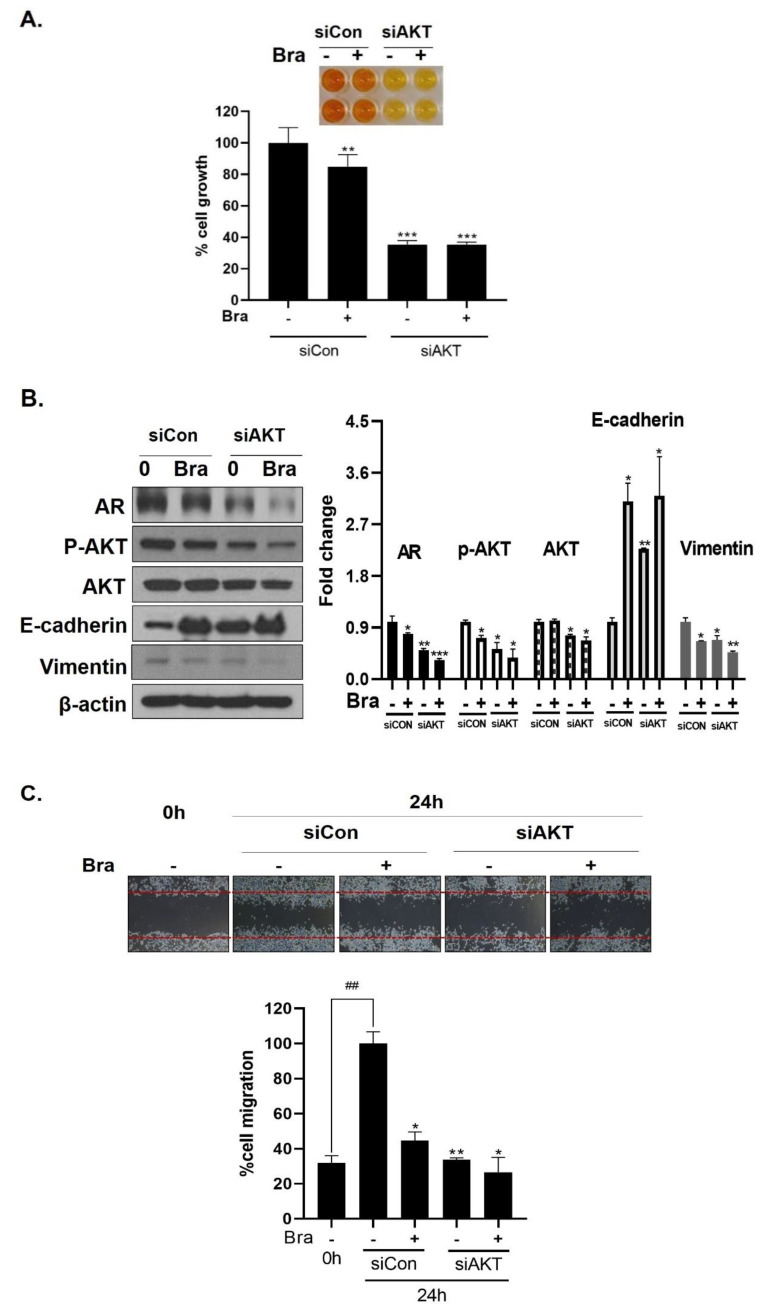

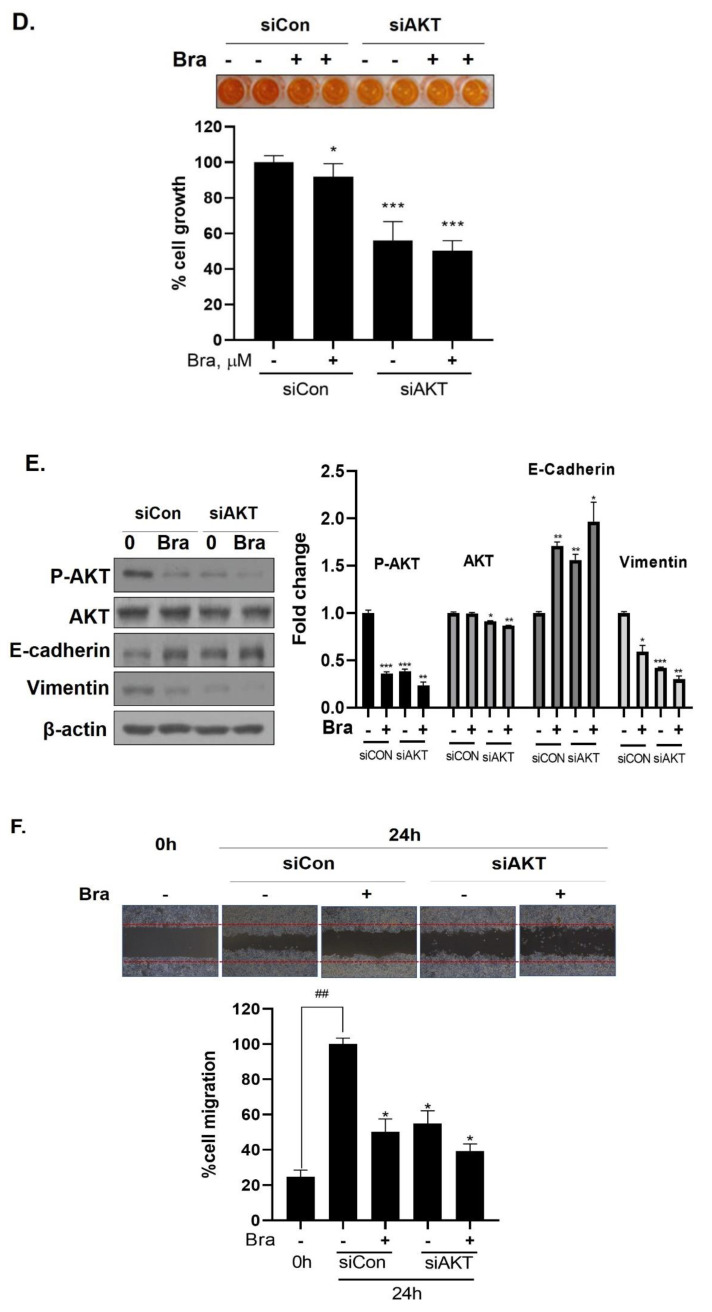
Effect of AKT siRNA on cell migration- and proliferation-related makers in Brassicasterol treated prostate cancer cells. (**A**–**B**) LNCaP cells were transfected with AKT siRNA for 48 h and were incubated in the presence or absence of brassicasterol (10 μM) for 24 h. (**A**) The cells growth was determined using Cellomax kit. The graph showed quantitative cell growth. (**) *p* < 0.01 and (***) *p* < 0.001 compared with control. (**B**) The cell lysates were prepared and subjected to Western blotting to determine the expression of AR p-AKT, AKT, E-cadherin, vimentin, and β-actin. Bar graphs represent the quantification of interest protein related to β-actin, present as a fold change of control of siRNA. (*) *p* < 0.05, (**) *p* < 0.01, and (***) *p* < 0.001 (in comparison to control of siRNA). (**C**) The cells were evaluated to cell migration by wound healing assay. Bar graph represents the quantification of cell migration, present as a percentage of control of siRNA. (*) *p* < 0.05, (**) *p* < 0.01 (in comparison to 24h control of siRNA) (##) *p* < 0.01 (in comparison to 0 h control). (**D**–**F**) PC-3 cells were transfected with AKT siRNA for 48 h and were incubated in the presence or absence of Brassicasterol (10 μM) for 24 h. (**D**) The cells were measured cell growth by Cellomax kit. The graph showed quantitative cell growth. (*) *p* < 0.05, and (***) *p* < 0.001 compared with control. (**E**) The cell lysates were prepared and subjected to Western blotting to determine the expression of p-AKT, AKT, E-cadherin, vimentin, and β-actin. Bar graphs represent the quantification of interest protein related to β-actin, present as a fold change of control of siRNA. (*) *p* < 0.05, (**) *p* < 0.01, and (***) *p* < 0.001 (in comparison to control of siRNA) (**F**) The cells were evaluated to cell migration by wound healing assay. Bar graph represents the quantification of cell migration, present as a percentage of control of siRNA. (*) *p* < 0.05(in comparison to 24 h control of siRNA) (##) *p* < 0.01 (in comparison to 0 h control).

As shown in Figure 5B, AR siRNA did not control p-AKT expression levels. To assess whether AKT mediated AR signaling pathway inhibitory effect by brassicasterol, we examined the AR signaling pathway regulation by AKT siRNA. AKT knockdown showed decreased AR (0.49-fold), AKT (0.76-fold), and p-AKT (0.52-fold) expression in LNCaP cells. In addition, AKT siRNA enhanced brassicasterol-induced AR inhibition (brassicasterol, 0.79-fold; brassicasterol + AKT siRNA: 0.33-fold) (Figure 6B). Furthermore, AKT siRNA showed inhibition of cell growth and increased cell growth inhibitory effect of brassicasterol (Figure 6A,C). Brassicasterol treatment showed 18% inhibitory effect on cell growth, but cell growth inhibition of brassicasterol was increased up to 65% by AKT siRNA (Figure 6A). Furthermore, AKT knockdown exhibited suppressed cell migration compared to control of siRNA. Consistent with cell growth data, brassicasterol treatment showed 54% inhibitory effect on cell migration, but cell migration inhibition of brassicasterol was increased up to 74% by AKT siRNA (Figure 6C).

### 3.7. Brassicasterol Exerts an Anti-Cancer Effect in AR-Independent Cancer as well as AR-Dependent Cells by Inhibiting AKT

We checked the expression of EMT markers, cell migration, and cell growth in PC-3 cells using AKT siRNA to assess whether the targeting AKT contributed to the anti-cancer effect in AR-independent prostate cancer cells, PC-3 cells. AKT siRNA decreased vimentin expression (0.68-fold) and increased E-cadherin expression (2.27-fold). Furthermore, AKT siRNA enhanced the brassicasterol EMT regulation effect (Figure 6E). Similar to LNCaP data, AKT siRNA inhibited cell growth and migration and contributed to inhibitory cell growth and migration effect of brassicasterol in PC-3 cells (Figure 6D,F). Brassicasterol treatment showed 9% inhibitory effect on cell growth, but cell growth inhibition of brassicasterol was increased up to 50% by AKT siRNA in PC-3 cells (Figure 6D). Furthermore, AKT knockdown exhibited suppressed cell migration compared to control of siRNA. Consistent with cell growth data, Brassicasterol treatment showed 50% inhibitory effect on cell migration, but cell migration inhibition of brassicasterol was increased up to 61% by AKT siRNA (Figure 6F).

## 4. Discussion

LNCaP cells are commonly used in studying androgen-sensitive early-stage prostate in oncology. LNCaP cells are a hormone-responsive cell line whose in vitro cell growth and acid phosphatase production are regulated by DHT [33]. As shown in Figure 1, *Hippocampus abdominalis* lipid extract (SHL) inhibited the elevated AR and PSA expression by DHT in LNCaP cells.

Phytosterols from *Hippocampus* species (*Hippocampus kelloggi* Jordan and Snyder, *Hippocampus histrix* Kaup, *Hippocampus kuda* Bleeker, and *Hippocampus trimaculatus* Leach) have been investigated in several studies [34,35]. However, phytosterols from *H. abdominalis*, has not been studied. Furthermore, brassciasterol from *Hippocampus* has not been investigated. Therefore, we checked brassicasterol in SHL. GC-MS analysis showed that brassicasterol was present in SHL (Figure 2A,B).

Brassicasterol was also tested to confirm whether it contributed to the anti-androgen effect of SHL. We found the brassicasterol decreased both AR mRNA and protein levels in LNCaP cells (Figure 3B,C). Further, brassicasterol decreased the upregulated AR protein level by DHT (Figure 3D). These data showed that brassicasterol had an anti-androgenic effect. According to Yazawa’s study, when brassicasterol was administered intraperitoneally to testosterone-treated castrated rats, the number of cell aggregates was reduced, which confirmed inhibition of bladder carcinogenesis [36]. Those data indirectly showed the anti-androgenic effect of brassicasterol.

Long-term treatment with brassicasterol showed potent cell growth inhibition even at low concentrations (Figure 4A). On the other hand, after short-time treatment, apoptosis was induced only at high concentration (50 μM) (Figure 4B). Moreover, cell growth inhibition of brassicasterol was also confirmed using 3D culture. The 3D culture model produces biochemical responses similar to parental tumors. Therefore, 3D cell culture model is applicable to predict in vivo therapeutic efficacy [37].

We found that the cancer cell motility and growth inhibition of brassicasterol was more enhanced after treatment with AR siRNA (Figure 5). However, the contribution of ARsiRNA to the effect of brassicasterol did not affect the regulation of AKT phosphorylation (Figure 5B). These data indicate that AR does not regulate AKT.

LNCaP cells shows a high constitutive AKT activity by lacking active lipid phosphatase PTEN, a negative regulator of the phosphatidylinositol (PI) 3-kinase/AKT pathway [38,39]. Activation of AKT is strongly correlated with prostate cancer. AKT pathway positively regulates protein synthesis, cell cycle, proliferation, invasion, metastasis, angiogenesis, and overall survival [40,41]. AKT and AR synergistic interaction in an in vivo prostate regeneration model [42] give evidence that PI3K/AKT and AR pathways can be linked mechanistically. The relationship between these factors affects the progress and development of prostate tumor growth. Many studies have demonstrated that the regulation of AR is downstream of activated AKT; thus, AKT upregulates AR levels in prostate cancer [43,44,45]. Therefore, we examined whether AKT regulated AR using AKT siRNA in LNCaP cells.

PC-3 cells do not respond to androgens, glucocorticoids, or fibroblast growth factors [46]. These cells are negative PTEN expression due to having a homozygous deletion of the PTEN gene; on the other hand, they show a high constitutive AKT activity [47]. Brassicasterol suppressed cell motility and growth by regulating AKT in both LNCaP and PC-3 cells (Figure 6).

Most prostate cancers have a loss of PTEN and highly constitutive AKT activation. Early anti-androgen treatment may be helpful in AR-dependent prostate cancer. However, continuous anti-androgen treatment causes CRPC with PI3K/AKT pathway activation [17]. Mulholland and Carver showed that both PI3K and AR pathway inhibition by AKT inhibitor and deprivation of androgen could head remarkable tumor reduction compared to inhibition of the single pathway [18,19].

EMT is associated with intracellular events including wound healing and cancer progression [48,49]. EMT is thought to play an important role in the development of both metastasis and therapy resistance [50].

AR and AKT are signaling pathways associated with EMT in prostate cancer. Elevation of AR expression and AR signaling promotes Pca metastasis by induction of EMT in prostate tumors [51]. AKT directly or in crosstalk with other signaling pathways can promote EMT [52,53]. We found AR and AKT siRNA induced E-cadherin and decreased Vimentin in LNCaP and PC-3 cells and mediated brassicasterol-induced suppression of EMT (Figure 6B and D).

Collectively, these data showed that brassicasterol from edible aquacultural *H. abdominalis* exerted an anti-cancer effect by dual-targeting AKT and AR signaling in prostate cancer.

## 5. Conclusions

*H. abdominalis* lipid extract (SHL) inhibited AR in DHT-induced LNCaP cells. GC-MS data showed that brassicasterol was present in the *H. abdominalis*. Brassicasterol inhibited AR and PSA in LNCaP cells. Brassicasterol inhibited cell growth and induced sub-G1 phase arrest in LNCaP cells. AR was associated with brassicasterol-induced suppression of cell proliferation, migration, and EMT in LNCaP cells. AKT-mediated the brassicasterol-induced suppression of the AR signaling pathway. Brassicasterol exerted an anti-cancer effect in AR-independent cancer as well as AR-dependent cells by inhibiting AKT.

Finally, we found that brassicasterol from *H. abdominalis* had an anti-cancer effect. In summary, brassicasterol from edible aquacultural *H. abdominalis* exerts an anti-cancer effect by dual-targeting AKT and AR signaling in prostate cancer.
